# Adapting Medication for Type 2 Diabetes to a Low Carbohydrate Diet

**DOI:** 10.3389/fnut.2021.688540

**Published:** 2021-08-09

**Authors:** Mark Cucuzzella, Karen Riley, Diana Isaacs

**Affiliations:** ^1^West Virginia University School of Medicine, Morgantown, WV, United States; ^2^Institute for Personalized Therapeutic Nutrition, Vancouver, BC, Canada; ^3^Cleveland Clinic, Cleveland, OH, United States

**Keywords:** low carb diet, diabetes, deprescribing, ketogenic diet, insulin resistance, metabolic syndrome, diabetes remission

## Abstract

Healthcare professionals in the primary care setting need to be competent to safely adapt diabetes medications when patients with Type 2 Diabetes (T2D) alter their diet. Safe prescribing practice is supported through an understanding of the clinical evidence, basic science, and pharmacology of medications. This review article supports clinicians in the practical application of this knowledge to achieve safe practice. Traditional medical training and clinical practice for chronic disease has long revolved around the teaching of intensifying therapy and evidenced based prescribing, a crucial skill when chronic disease progresses. Now that we are witnessing remission of Type 2 Diabetes through nutritional interventions specifically low carbohydrate diets (LCD) we must apply the same effort and thought to de-prescribing as the underlying metabolic condition improves. There is minimal guidance in the literature on how to actively de-prescribe. The American Diabetes Association in their *Standards of Medical Care in Diabetes–2021* acknowledges low carbohydrate nutritional therapy (LCD) as a viable option in the management of Type 2 Diabetes (T2D). Thus, the goal of our paper is to help close the gap between the clinical evidence, basic science, and pharmacology of T2D medications to the practical application and teamwork needed to facilitate safe medication reduction in the primary care setting when applied to a LCD. The LCD is an increasingly popular and effective option for managing T2D and can lead to an improvement in the condition, reduced medication burden, and contribute to significant weight loss. Safe initiation of a LCD in patients on medications requires significant monitoring and medication adjustments to decrease and eliminate the risk of hypoglycemia and hypotension. The health care team including clinicians in primary care, nursing, pharmacy and nutrition need to be competent in adjusting diabetes and antihypertensive medications to achieve safe and effective care. The most immediate and important adjustments are to insulin, sulfonylureas, SGLT2 inhibitors, blood pressure medications and diuretics. Interdisciplinary care teams can individualize therapy while following the guidance, which includes monitoring blood glucose and blood pressure closely, decreasing medications that can cause hypoglycaemia and hypotension, evaluating blood glucose and blood pressure data responses regularly, and open access communication with the team. The article is an international consensus document on de-prescribing that was put together by a multidisciplinary team of clinicians.

## Introduction

Healthcare professionals in the primary care setting need to be competent to safely adapt diabetes medications when patients with Type 2 Diabetes (T2D) alter their diet. Safe prescribing practice is supported through an understanding of the clinical evidence, basic science, and pharmacology of medications. This review article supports clinicians in the practical application of this knowledge to achieve safe practice.

The American Diabetes Association (ADA) in their *Standards of Medical Care in Diabetes 2021* recognize low carbohydrate nutritional therapy as a viable option for the management of T2D. The publication states “For people with type 2 diabetes, low-carbohydrate and very-low-carbohydrate eating patterns, in particular, have been found to reduce HemoglobinA1C (HbA1c) and the need for antihyperglycemic medications” (1). In addition the *Nutrition Therapy for Adults with Diabetes or Prediabetes: A Consensus Report 2019* states: “Reducing overall carbohydrate intake for individuals with diabetes has demonstrated the most evidence for improving glycemia and may be applied in a variety of eating patterns that meet individual needs and preferences. For individuals with type 2 diabetes not meeting glycemic targets or for whom reducing glucose-lowering drugs is a priority, reducing overall carbohydrate intake with a low- or very-low-carbohydrate eating pattern is a viable option” (2). The report adds, “Use of organization-approved protocols for insulin and other glucose-lowering medications can help reduce therapeutic inertia and/or reduce the risk of hypoglycemia and hyperglycemia” (2). Furthermore, the 2019 report states “Low-carbohydrate eating patterns, especially very low-carbohydrate (VLC) eating patterns, have been shown to reduce A1C and the need for antihyperglycemic medications. These eating patterns are among the most studied eating patterns for type 2 diabetes” (2). The ADA publications are mirrored internationally by the European Association for the Study of Diabetes (EASD) (3), and Diabetes Canada (4).

Clinical experience finds that a low carbohydrate diet (LCD) can be effective for all forms of diabetes mellitus, including T2D, and those characterized by a low insulin state such as Type 1 Diabetes (5). This review will only discuss medication adaptation for T2D. It should be noted that rapid physiologic changes can be expected and close monitoring with timely communication of glucose and medication management is essential to ensure patient safety and optimial efficacy. Potential harms include hypoglycemia due to insulins, and insulin secretagogues, and ketoacidosis due to SGLT2 inhibitors. Equally it is important to consider that carbohydrate restriction should be tailored to the specific needs and health goals of the person living with diabetes.

There is a growing body of published literature discussing the clinical application of a LCD for T2D. Virta Health's report on their novel digitally-monitored continuous care intervention at 2 years demonstrated sustained long-term beneficial effects on multiple clinical markers of diabetes and cardiometabolic health while utilizing less medication (6). In a primary care setting in United Kingdom, Dr. David Unwin published his patient data over 6 years (7). The findings from Unwin et al. include: (1) For those choosing a lower carbohydrate dietary approach for an average of 23 months it is possible to achieve a 46% drug-free T2D remission rate in UK primary care while also achieving significant improvements in weight, blood pressure and lipid profiles; (2) in patients with prediabetes, a LCD approach reduced HbA1c to within a non-diabetes threshold in 93% of patients; (3) participants who started with the highest HbA1c saw the greatest improvements in glycemic control (7). These clinical findings of a LCD compare favorably to historical usual care for T2D. The usual-care control arm of the DiRECT Trial achieved <2% achieved A1C <6.5% (8).

Achieving tight glycemic targets is important for preventing microvascular complications such as neuropathy, nephropathy, and retinopathy. However, modern treatment of T2DM using pharmacological approaches does not consistently achieve HbA1c targets. Higher HbA1c is associated with more diabetes complications, morbidity, and mortality (9). Lowering HbA1C alone does not always reduce complications. The Action to Control Cardiovascular Risk in Diabetes (ACCORD) trial demonstrated that intensive medical treatment carries an increased risk of all-cause mortality, a 35% increased risk of cardiovascular mortality, and a greater risk of hypoglycemic events and weight gain of 10 kg compared to those on standard insulin therapy (10). Other multinational, multicenter, randomized controlled trials that used medications to achieve tight glycemic targets did not demonstrate the expected reductions in heart disease or in overall mortality (11–16). There is strong evidence for an alternative approach to treating people with T2DM.

Despite the acceptance of reducing carbohydrates as a powerful option in the T2D management there is still a certain amount of clinical inertia and a large gap between the awareness of the benefits of this intervention and the practical application. Even in the recent 63 page publication in the Lancet from the Lancet Commission on Diabetes that embodies 4 years of extensive work to make recommendations to improve clinical practice, carbohydrates are only mentioned once and only in relation to adjusting insulin doses (17).

## The Low Carbohydrate Diet in Type 2 Diabetes

Dietary carbohydrate restriction and LCD has lacked a consistent definition and has been used to refer to carbohydrate intake levels that are low only in relation to population averages, often measured as a percentage of kcals, but do not reach the therapeutic levels of restriction necessary to address insulin resistance and T2D. In some reported studies, a LCD has included up to 45% of daily calories from carbohydrates. For an individual consuming 2,500 calories a day this would be 280 g of carbohydrates. A LCD by some definitions comprises <130 grams of digestible carbohydrates per day which is <50% of the average daily intake in the UK and US. Reduction to levels below 50 grams of digestible carbohydrates a day are often needed to fully address insulin resistance and promote T2D remission. Digestible carbohydrate is defined as simple sugars and complex carbohydrates such as starch, which is digested to glucose; this is in contrast to fiber, which is a carbohydrate that is not digested or is only partly digested with the aid of intestinal bacteria. Recommended food choices on a lower carbohydrate meal plan include (1) non-starchy vegetables, (2) protein-containing foods such as fish, meat, poultry, and eggs, (3) natural fats such as olive oil and butter and (4) foods that naturally contain fats, fiber, and/or protein such as nuts, olives, and avocado. Sugar and refined, starchy carbohydrates are eliminated or greatly reduced.

### Defining Therapeutic Carbohydrate Reduction and Low Carbohydrate Diets (LCD)

There are many ways to implement dietary carbohydrate reduction. The following represents some proposed definitions that represent the variety of therapeutic approaches including in carbohydrate reduction. These are based on protocols currently in use and on definitions found in the literature (18–22):

VLCK (very low-carbohydrate ketogenic) meal plan recommend 30 g or less of dietary carbohydrate per day without restriction of kilocalorie (kcal). Instead, VLCK and LCK diets rely upon satiety to guide caloric needs.

LCK (low-carbohydrate ketogenic) meal plans recommend 30–50 g of dietary carbohydrate per day without restriction of kcals. Sometimes “net carbs” (calculated by total carbohydrate minus fiber) will be used with a goal of 25–30 g net carbs/day.

RC (reduced-carbohydrate) meal plans recommend at least 50 g, but <130 g of dietary carbohydrate per day, a level that is higher than therapeutic levels listed above and lower than the U.S. Institute of Medicine dietary reference intake (DRI) for carbohydrate. Restriction of kcals may or may not be recommended at this level.

MCCR (moderate-carbohydrate, calorie-restricted) meal plans recommend more than 130 g of dietary carbohydrate per day with a range of 45–65% of daily kcals coming from carbohydrate (18). In most cases, kcals are also restricted to maintain energy balance or to or promote weight loss. This dietary intervention reflects the amount of dietary carbohydrate typically found in the “*carbohydrate counting” dietary intervention that is given to many people with T2DM*.

This article follows common practice in using the term “low-carbohydrate diet” or LCD to refer to a variety of carbohydrate-reduction therapies implemented in clinical settings that fall below 130 g of dietary carbohydrate per day. However, the specific protocol under discussion here is a LCK diet. Clinicians should note that other interventions for remission of T2DM, such as very low-calorie diets or intermittent fasting, effectively reduce carbohydrate intake as part of overall kcal reduction. Conversely, reducing carbohydrate intake in practice often serves to reduce overall kcal. Recommendations for kcal restriction or “calorie counting” are not typically part of VLCK and LCK clinical interventions, but may be used in research protocols.

Immediate diabetes and even blood pressure medication reduction or elimination is standard care in patients undergoing gastric bypass. The practice is similar in patients undergoing a very low calorie low carbohydrate protocol. An example of this was in the DiRECT trial that required: “All oral antidiabetic and antihypertensive drugs to be discontinued on day 1 of the weight management programme, with standard protocols for drug reintroduction under national clinical guidelines, if indicated by regular monitoring of blood glucose and blood pressure. The clinical emphasis must be on glucose and blood pressure monitoring especially in the first weeks” (8).

Other examples of studies are listed in Table 1 that provide some guidance on the medication adjustments and frequency of monitoring for diabetes and antihypertension medications.

**Table 1:** Medication adjustments for LCD meal plans summary of studies.

**Study**	**Type of medication**	**Adjustments made**	**Frequency of monitoring for medication adjustments**
Yancy et al. (19) (*n* = 21)	Insulin	Reduced 50% upon starting diet.	Every other week for 16 weeks
Single-arm pilot intervention trialType 2 diabetes	Sulfonylureas	Reduced 50% or discontinued upon starting diet.	
	Diuretics	Reduced 50%. Discontinued if on low dose (25 mg of hydrochlorothiazide or 20 mg of furosemide) upon starting diet.	
Westman et al. (20) (*n* = 49)Prospective observational studyType 2 diabetes	Insulin	Discontinued or reduced from 12.5 to 90% at 24 weeks	Weekly for 3 months, then every other week for 3 months
Salow et al. (21) (*n* = 16)Parallel-group randomized trialType 2 diabetes and prediabetes	Sulfonylureas/DPP-4 inhibitors	All participants discontinued medications by 12 months post-baseline	Not specified
Athinarayanan et al. (6) (*n* = 267)Open label, non-randomized, controlled studyType 2 diabetes	Insulin Sulfonylureas	Total insulin reduced 62%Sulfonylureas reduced 100%	Continuous Care Intervention with remote patient monitoring
Saslow et al. (22) (*n* = 16)Parallel-group randomized trialType 2 diabetes and prediabetes	Sulfonylureas	Doses were reduced 50% if the entry HbA1c was <7.5%; discontinued if pre-dinner glucose levels went below 110 mg/dL in spite of prior dose reduction	Participants were asked to monitor fasting glucose and before dinner daily. Doctor visits unspecified.
	Thiazolidinediones	Discontinued for those with starting HbA1c below 7%.	
	Metformin	Continued	
Yabe et al. (18) (*n* = 24)Randomized, open-label, 3-arm parallel comparative exploratory studyType 2 diabetes	SGLT2 inhibitors	N/A. Discontinuation recommended.	Weekly
Unwin et al. (7) (*n* = 54 on DM meds)ongoing audit of service provision	Sulfonylureas, Insulin, Metformin,SGLT2i, GLP1a	Discontinued completely in 19 (35%)	Regular office visits w GP offices
Lean et al. (8) DiRECT (*n* = 148)111 on DM meds) 12 mos.Open-label, cluster-randomized trial	Not specified	Approx 75% taking meds both groups at start. 74% off all meds intervention group, 18% controls.	Regular office visits w GP offices

## Categories of Drugs Used in T2DM Patients-Specific Medications and Mechanisms

A brief summary of various agents used for diabetes and their mechanisms and adverse effects are provided below (1, 3, 4).

1. **Biguanides**–Metformin is the only drug in this class. Metformin reduces liver glucose output and slightly lowers insulin resistance in muscles and adipose tissue, and can decrease intestinal glucose uptake. Advere effects can include gastrointestinal side effects such as diarrhea, vomiting and abdominal pain, vitamin B12 deficiency, worsening of neuropathic symptoms. Side effects can be mitigated with extended release preparation. Metformin has numerous beneficial pleitropic actions, the consideration of which is outside the scope of this article.
2. **Sulfonylureas** include glyburide, glibenclamide, glipizide, glimiperide, gliclazide. Sulfonylureas stimulate the pancreas to secrete more insulin. Adverse effects include hypoglycemia, weight gain and potential pancreatic beta cell failure.
3. **DPP-IV inhibitors** include sitagliptin, saxagliptin, linagliptin, alogliptin. These agents prevent the breakdown of GLP-1 hormone which lowers glucagon, increases insulin, slows gastric emptying, and reduces the appetite in a glucose dependent manner. These medications have less A1C lowering compared with GLP-1 receptor agonists. Infrequent adverse effects are abdominal pain, diarrhea, nausea and headache.
4. **Thiazolidinediones (TZD)** include pioglitazone and rosiglitazone. These agents improve insulin resistance, but can contribute to weight gain due to insulin sensitivity in the adipose tissue. They are associated with many adverse effects such as peripheral edema, osteoporosis, heart failure as well as a risk of new primary bladder cancer.
5. **Meglitinides** include nataglinide and repaglinide. These cause the pancreas to release more insulin. Similar to sulfonylureas but with a shorter half-life, they more frequent dosing and have slightly lower risk of hypoglycemia.
6. **Alpha glucosidase inhibitors** include acarbose and miglitol. They prevent absorption of carbohydrates and can cause gastrointestinal symptoms including gas and bloating.
7. **Sodium-Glucose Transporter 2 Inhibitors (SGLT2i)** include canagliflozin, dapagliflozin, empagliflozin, and ertugliflozin. These agents prevent the kidneys from absorbing glucose back into the bloodstream so more is excreted in the urine. They can cause euglycemic diabetic ketoacidosis (even in T2DM patients), genital bacterial and fungal infections, dehydration and hypotension. Several agents in this class have been shown to reduce the progression of chronic kidney disease and to have cardioprotective effects.
8. **Glucagon Like Peptide-1 receptor agonists (GLP1-RA)** include exenatide, liraglutide, dulaglitide, semaglutide, and lixisenatide. They increase GLP-1 which leads to a glucose dependent increase in insulin and decrease in glucagon which leads to decreased glucose including postprandial glucose. They also delay gastric emptying and enhance satiety which helps facilitate weight loss. They are more potent than DPP-4 inhibitors. Adverse effects include gastrointestinal side effects such as nausea, vomiting, diarrhea, and pancreatitis. They are contraindicated if a patient has a personal or family history of medullary thyroid cancer.
9. **Basal insulins** include insulin glargine, insulin detemir, insulin glargine U300, Degludec U100, U200, Humulin U-500, and NPH. Insulin stimulates glucose to be taken up by muscle, liver, fat cells, and brain tissue. Insulin also inhibits glucagon action so is anti-catabolic. Supra-physiologic doses in those with insulin resistance may increase hunger and contribute to weight gain. Adverse effects include hypoglycemia and lipodystrophy.
10. **Bolus insulins** include Regular, Lispro, Aspart, Glulisine, lispro-aabc, and inhaled insulin. Effects are similar to basal insulin but they are shorter acting and ware off more quickly.
11. **Amylin Mimetics** include pramlintide which is less commonly used since it requires multiple daily injections in addition to meal-time insulin. Pramlintide is a synthetic hormone that resembles human amylin, a hormone that is produced by the pancreas and released into the blood after meals where it helps the body to regulate levels of blood glucose. Amylin slows the rate at which food (including glucose) is absorbed from the intestine and reduces the production of glucose by the liver by inhibiting the action of glucagon. Adverse effects include nausea, vomiting, and hypoglycemia.
12. **Other agents with T2D indications**. Bromocriptine and bile acid sequestrants have also been used with some efficacy in management of T2D.

## Diabetes Medications and a Low Carbohydrate Diet

Diabetes medications work by different mechanisms of action and vary based on their benefits and risks. This is important to consider in light of new evidence for various diabetes agents. A summary table of risks to benefits can be found in Table 2.

**Table 2:** Summary of type 2 diabetes medication benefits/risks (1, 23, 24).

	**Biguanides**	**Secretagogues**	**DPP4is**	**GLP1RAs**	**SGLT2i**	**TZD**	**Insulin**
Agents	Metformin	Sulfonylureas Glyburide Glibenclamide Glipizide Glimperide Gliclazide Megitinides Nateglinide Repaglinide	Sitagliptin Saxagliptin Linagliptin Alogliptin	Exenatide Liraglutide Dulaglutide Lixisenatide Semaglutide	Canagliflozin Dapagliflozin Empagliflozin Ertugliflozin	Pioglitazone Rosiglitazone	Rapid-acting insulin Basal insulins
Mechanism of action	Decreases hepatic gluconeogenesis, decreases intestinal absorption of glucose, improves insulin sensitivity by increasing peripheral glucose uptake	Stimulates pancreatic islet cells which causes an increase in insulin secretion. These drugs are not effective in the absence of functioning beta cells	Dipeptidyl peptisase-4 inhibitor that slows the inactivation of incretin hormones GLP-1 and GIP	Increase GLP-1 which leads to glucose dependent ↑ in insulin & ↓ in glucagon and ↓ glucose. Benefts of delayed gastric emptying and enhanced satiety	Sodium-glucose co-transporter 2 inhibitor that reduces reabsorption of filtered glucose and lowers the renal threshold for glucose thereby increasing urinary glucose excretion	Thiazolidine- dione that enhances insulin sensitivity in adipose tissue, skeletal muscle and liver to decrease plasma glucose, insulin concentrations	
Hypoglycemia	Neutral	Moderate-severe (^*^mild glinide)	Neutral	Neutral	Neutral	Neutral	Moderate to severe
Weight	Slight loss	Gain	Neutral	Loss	Loss	Gain	Gain
Renal/GU	Not for eGFR <30	Hypoglycemic risk	Renal dosing except linagliptin	No exenatide for CrCl <30	Reduce progression of CKD	Neutral	More hypoglycemia risk
GI ADR	Moderate	Neutral	Neutral	Moderate	Neutral	Neutral	Neutral
Cardiac/CHF	Neutral	More CHF risk	Possible with saxagliptan, alogliptan	Possible benefit liraglutide	Reduced risk hospitalization for heart failure	Moderate	More CHF risk
Cardiac/ASCVD	Neutral	Risk↑	Neutral	Benefit liraglutide, semaglutide, dulaglutide, albiglutide	Benefit empagliflozin, canagliflozin	May reduce stroke risk	Neutral
Bone	Neutral	Neutral	Neutral	Neutral	Canagliflozin warning	Moderate fracture risk	Neutral
Ketoacidosis	Neutral	Neutral	Neutral	Neutral	DKA in T2D in stress	Neutral	Neutral

* *Mild with glinide; Mod/severe with sulfonylurea. DPP4is, Dipeptidyl Peptidase 4 Inhibitors; GLP1Ras, Glucagon-like-peptide-1 receptor agonists; SGLT2i, sodium glucose cotransporter 2 inhibitors; TDZ, Thiazolidinediones. ↓, decrease; ↑, increase*.

What is most significant with diabetes medications and a LCD is that blood glucose levels typically fall rapidly and substantially when an individual adopts a LCD. It is therefore essential that medications are adjusted in order to prevent hypoglycemia. The following recommendations are based on combined clinical expertise, clinical trials, and from current published guidelines on LCD and medications.

When deciding the safety and appropriateness of T2D medications with a LCD there are three key clinical considerations:

° Is there a risk of the drug causing hypoglycemia or other adverse events?
° What is the degree of carbohydrate restriction?
° Once carbohydrates are reduced, does the drug continue to provide health benefit, and if so, are the potential benefits greater than or less than the possible risks and side effects?

**Figure 1:**
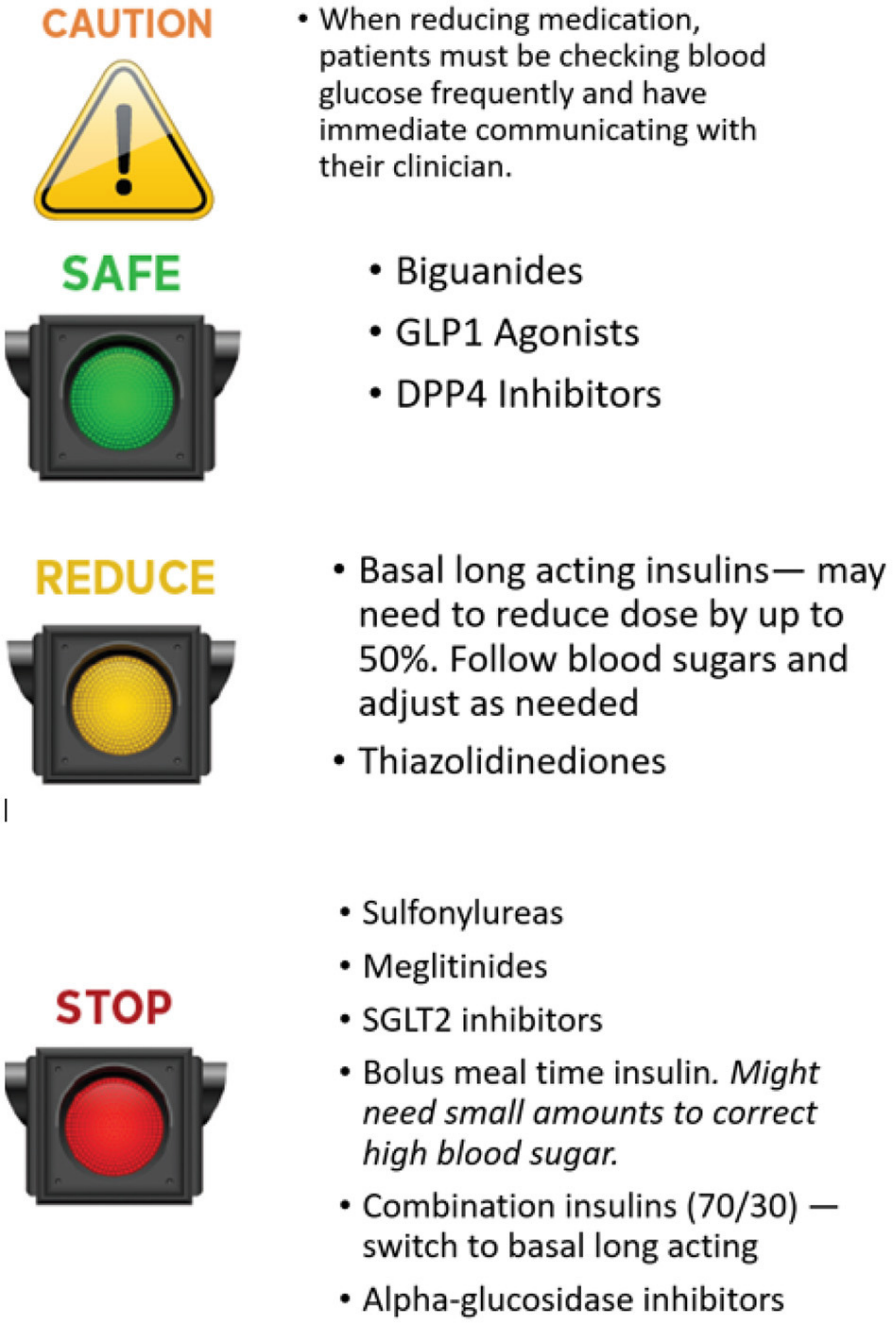
Stoplight approach to medication management with therapeutic carbohydrate reduction.

The preferences of the person with diabetes should be taken into account in all decisions on medication changes. Clinicians must support patients by balancing the pros and cons of different approaches. Cost is an issue that may influence medication choice in many health care systems. Medication costs are a large burden to individuals and the health systems so it is advisable before prescribing expensive medication that all other options have been considered. Cardiovascular and renal benefits of certain medications should be taken into consideration and may warrant continued use even when a person has met their A1C target. An easy approach to consider when adjusting medications is using a stop light approach (see Figure 1).

### Medications That Create a Risk of Hypoglycemia

A risk of hypoglycemia exists with sulfonylureas, meglinitides, and exogenous insulins. When carbohydrate intake is reduced, these medications need to be reduced or stopped, with adjustment being individualized to patient circumstances. The authors recommend at least a 50% reduction in dose of insulins while stopping the sulfonylureas and meglinitides. Further reductions in insulin may be necessary according to the blood glucose response. There may be a period of short term hyperglycemia while the individual adapts to a lower carbohydrate intake; this is preferable to the risk of hypoglycemia from not reducing doses. These patients benefit from reducing hyperinsulinemia as this is thought to contribute to many of the metabolic and other abnormalities seen in type 2 diabetes. Hypoglycemia can contribute to increased hunger, making it more difficult to lose weight (25).

#### Sulfonylureas and Meglitinides

The absence of long-term health benefits of these drugs provides reassurance that stopping them will not adversely affect long term health. Sulfonylureas as second line drugs may increase risk of myocardial infarction, all-cause mortality, and severe hypoglycemia especially in the elderly, compared with remaining on metformin monotherapy (26). Glucose variability and glucose spikes may also be associated with increased cardiovascular risk (27, 28).

#### Insulins

Unless embarking on an 800 kcal/day very low calorie and low carbohydrate diet similar to DiRECT, practical expertise suggests a 50% reduction of total daily insulin dose at initiation of the LCD is appropriate in most cases. In individuals whose HbA1c is markedly elevated, a smaller reduction (e.g., of 30%) may be appropriate, with further reductions over time. In individuals on a basal bolus regimen, it is advised to preferentially reduce or stop bolus insulin. As glucose levels improve, basal insulin can then be reduced. Mixed insulin should be stopped and switched to basal insulin alone and the daily dose can be reduced by 30–50% at the start of LCD. If on a single dose of long-acting insulin with a peak, the preferred timing is to administer in the morning to coincide with higher insulin levels with normal circadian physiology of daytime feeding and a reduction of circulating insulin with a nighttime fast. Many basal insulins do not have a high peak especially with reduced doses, allowing it to be administered at any time of day. Some patients can expect to eliminate the need for insulin completely, over days or months, as insulin resistance resolves.

It should be cautioned that some people diagnosed with T2D may in fact have an insulin deficiency form of diabetes, such as Latent Autoimmune Diabetes of Adults (LADA) or Maturity Onset Diabetes of Youth (MODY). These patients should not have their insulin stopped completely. Endogenous insulin insufficiency is more likely in patients who were not overweight at diagnosis of diabetes or required insulin earlier in their course of diagnosis (29). They are also likely to be more insulin sensitive, requiring smaller insulin doses than typically used in T2D. Over-reduction in insulin dosage in these patients would lead to significant hyperglycemia, and further dosage reduction should be avoided. It is recommended that expert advice and additional testing such as c-peptide and GAD antibodies is sought in cases of doubt.

### Medications That Increase Ketoacidosis Risk

#### SGLT2 Inhibitors

These medications carry a risk of euglycemic ketoacidosis. A LCD alone cannot cause ketoacidosis, but it may enhance the risk posed by SGLT2i's by lowering insulin levels because insulin inhibits ketone formation. SGLT2i-induced ketoacidosis may occur with normal blood glucose levels, and this heightens the risk of ketoacidosis going unrecognized. It is worth noting that a very low carbohydrate diet (typically <50 g of carbohydrate a day) can produce a physiologically normal state of ketosis, that should not be confused with the pathological state of diabetic ketoacidosis. Despite recent literature supporting slight cardiovascular risk reduction and renal protection of SGLT2i's, it is recommended that SGLT2i's are used with caution in those adhering to a low carbohydrate eating plan. It is appropriate to stop SGLT2i's in many cases, particularly in those adhering a very low carbohydrate diet (30–50 g/day). A GLP-1 agonist is a safer choice as a second-line agent after metformin. An excellent review of the physiology of a LCD mimicking many effects of SGLT2i was recently pubished by Murray et al. (30).

### Medications With Minimal Risk but Little to No Benefit

#### Thiazolidinediones

These agents are safe to continue from a short-term perspective as they do not cause hypoglycemia. Concerns exist over their long-term safety, including risks of bladder cancer (31), heart failure (32), and reduced bone mineral density (33). It is recommended to stop thiazolidinediones as soon as glucose levels allow. Thiazolidinediones are also know to cause weight gain (34).

#### Acarbose

Although acarbose is safe to continue whileon commencing on a LCD, the benefits are much less pronounced because of reduced starch ingestion so the patient can usually stop the medication.

### Medications That Pose No Excess Risk With a LCD and May Have Benefit

***Metformin***is safe to continue and in some patients continues to offer favorable benefits. There is no hypoglycemia associated with metformin and neutral or minor weight loss. However, up to 25% of people experience gastrointestinal side effects from metformin (35).

***GLP-1 agonists***: Safe to continue. Benefits with a LCD include increased satiety and slowed gastric emptying (36) and cardiovascular benefits (37). With a sustained LCD, people may be able to stop their GLP-1 agonist. However, guidelines encourage continued use for those with Atherosclerotic Cardiovascular Disease (ASCVD) or high ASCVD risk independent of A1C. For a detailed review of the multiple mechanisms of this class of medications refer to the full review from Drucker (38).

***DPP4 inhibitors***are less potent than GLP-1 agonists but safe to continue as they do not cause hypoglycemia and are weight neutral. Clinical experience from the International group of authors agreed these seem to have little blood glucose lowering effect in the context of a LCD.

A summary of medication adjustments for diabetes can be found in Table 3. Appendix 1 is a list of current published clinical guidelines on LCD with medication reduction suggestions.

**Table 3:** Type 2 diabetes: diabetic medications on a low carbohydrate diet– a summary and suggestions (36).

**Drug group**	**Action**	**Hypo risk?**	**Suggested action (to continue/stop)**
Sulfonylureas	Increase pancreatic insulin secretion	Yes	STOP (or if gradual carbohydrate restriction then wean by e.g., halving dose successively)
Insulins	Exogenous insulin	Yes	REDUCE/STOP (Change to basal only and wean appropriately, e.g., successive 30–50% reductions, toward elimination) ^*^see below
Meglitinides	Increase pancreatic insulin secretion	Yes	STOP (or if gradual carbohydrate restriction then wean by e.g., halving dose successively)
Biguanides	Reduces insulin resistance	No	Optional, consider clinical pros/cons.
GLP-1 agonists	Slow gastric emptying. Glucose dependent pancreatic insulin secretion.	No	Optional, consider clinical pros/cons (expensive).
SGLT-2 inhibitors	Increase renal glucose secretion	No	Usually stop. Concern over possible risk of ketoacidosis (though this risk may be with LADA that has been misdiagnosed as T2DM). Use in selected patients may be beneficial in early reversal.
Thiazolidinediones	Reduce peripheral insulin resistance	No	Usually stop. Concern over risks usually outweighs benefits.
DPP-4 inhibitors	Inhibit DPP-4 enzyme	No	Stop. No significant risk, but no benefit in most cases.
Alpha-glucosidase inhibitors	Delay digestion of starch and sucrose	No	Stop. No benefit on a low carbohydrate diet.

*There are three considerations with the use of diabetic medications in type 2 diabetes when on a low carbohydrate diet*.

*• Is there a risk of hypoglycaemia?*

*• What is the degree of carbohydrate restriction?*

*• Does the medication provide any benefit, and if so, do any potential benefits outweigh any side effects and potential risks?*

* *Insulin reduction suggestion Tailor to individual. If using basal-bolus regime convert to long-acting insulin only, BD in equal doses (OD may suit some people). If a very low carbohydrate diet is planned any bolus insulin dosing can simply be eliminated. On commencing low carbohydrate diet reduce insulin by 30–50%. Monitor QDS initially for hypoglycaemia (rescue glucose if required). Continue down-titration of insulin as insulin resistance improves (can take months). Goal for most can be to eliminate insulin*.

*Caution: Some people with T2DM may have pancreatic insufficiency. Also people with other forms or pancreatic insufficiency (e.g., LADA or T3c) may have been misdiagnosed as T2DM. Consider this if rapidly increasing HbA1c, thirst, polydipsia, weight loss, low C-peptide. Insulin should not be eliminated in this cohort*.

### Individualization of Therapy and the Role of Blood Glucose Monitoring

For those individuals who wish to adopt a very low carbohydrate or ketogenic diet (<50 grams of carbohydrate/day), a significant reduction or complete discontinuation of insulin may be required. Self-monitoring of blood glucose or continuous glucose monitoring (CGM) can be very helpful in providing rapid feedback on how foods affect blood glucose as a person adopts a LCD, and to inform whether medication doses can be reduced further. There is evidence that frequent paired glucose testing is effective in supporting appropriate food choices, regardless of the type of diabetes treatment (39, 40). Patients on drugs that increase the risk hypoglycemia should have access to rescue therapies (glucose tablets/gel or glucagon), an adequate supply of testing strips, and immediate access to a member of the health care team. This is especially important at the initiation of carbohydrate reduction. Checking blood glucose for the purpose of feedback and behavior change can be extremely effective (41).

It can be highly educational for patients to see their own glycemic response to food correlated to how they feel. This is now possible with continuous glucose monitoring (CGM) technology and has recently become more accessible and affordable with improved CGM technology. Such systems can show the large post-meal glucose spikes and increased glucose variability that are common in patients who have a standard high carbohydrate dietary patterns, are insulin-resistant, and in later stage T2D with beta cell insufficiency. The CGM can also show the impact of a LCD on reducing glucose spikes after meals (41, 42).

### Anti-hypertensive Medication Adjustment

It is important to review the medication list for anti-hypertensives. Blood pressure will need to be monitored either at home or in the clinic during initiation of the dietary intervention. Patients should be shown how to self-monitor blood pressure and be made aware of symptoms of low blood pressure, such as light-headedness upon standing or severe fatigue. These symptoms and/or systolic blood pressure below 120 should prompt reduction of anti-hypertensive medication. Hyponatremia may be exacerbated by SGLT2is, thiazides, or loop diuretics. The initiation of the diet is associated with diuresis and natriuesis; therefore, adequate sodium intake is emphasized to prevent dehydration and hypotension. From a recent review in J Hypertension, they stated that from “Our analysis suggests that insulin plays a primary role in hypertension, highlighting the tight link between essential hypertension and diseases associated with the metabolic syndrome” (43).

Boullion or broth with sodium is a good remedy as well as preventive measure in the initiation phase of the diet. Some patients with heart failure are salt sensitive so monitor this subgroup closely or reduce diuretics judiciously instead of advising sodium rich broths or foods. Tailor any reduction in anti-hypertensives to the patient's co-mordidities. Results of Dr David Unwin's 6 year observational trial showed a 10.9 mmHG reduction in systolic blood pressure (SBP) and 6.3 mmHG diastolic blood pressure reduction despite a 20% reduction in anti-hypertension medication (44).

### Other Medications Needing Adjustment

If there is a significant change in intake of leafy greens or other foods containing vitamin K, vitamin K antagonists (i.e., warfarin) will need frequent monitoring. Improvements in heartburn (gastroesophageal reflux disease) may allow reduction or elimination of proton pump inhibitors (PPIs) or H2 blockers. Diarrhea predominant irritable bowel syndrome may also improve with more bioavailable food containing essential proteins and fats. Patients with polycystic ovary syndrome (PCOS) may see an improvement in their condition with a return of fertility so advice on contraception may be required. Migraines and inflammatory joint pains also may improve and require medication adjustment. Due to the natural diuresis which occurs with insulin reduction and glycogen depletion, if loop diuretics are given for edema these can be safely reduced or removed as the edema is monitored.

### Other Lifestyle Interventions to Affect Insulin Resistance and Aid in Medication Reduction

Physical activity of all forms can assist insulin sensitivity. This can be just the general movement of walking and having an active day, to directed aerobic type activity, as well as high intensity activity and strength training. Adequate sleep, reducing stress, emerging data on the microbiome, time restricted eating, Vitamin D status, genetics, and multiple other modulators of insulin resistance and sensitivity are now being discovered and individually tailored by clinicians and patients. Discussion of these topics is beyond the scope of this paper.

## Discussion

The LCD is an increasingly popular and effective option for managing T2D and can lead to an improvement in the condition, reduced medication burden, and contribute to significant weight loss. A recent qualitative review reports medication reduction to be a primary reason for patients to start a LCD, even more important than weight loss (45). Safe initiation of a LCD in patients on medications requires significant monitoring and medication adjustments to decrease and eliminate the risk of hypoglycemia and hypotension. The health care team including clinicians in primary care, nursing, pharmacy and nutrition need to be competent in adjusting diabetes and antihypertensive medications to acheve safe and effective care. The most immediate and important adjustments are to insulin, sulfonylureas, SGLT2iss, blood pressure medications and diuretics. Interdisciplinary care teams can individualize therapy while following the guidance above, which includes monitoring blood glucose and blood pressure closely, decreasing medications that can cause hypoglycemia and hypotension, evaluating blood glucose and blood pressure responses regularly, and open access communication with the team.

Medical education and practice for decades has focused almost exclusively in prescribing and intensifying medical therapy as chronic disease progresses. Our international team of clinician authors hope for a day when medical education and practice will spend as much time, thought, and effort into safely de-prescribing medications as our patients restore health.

## Author Contributions

International group all contributed to the piece with MC, KR, and DI. All authors contributed to the article and approved the submitted version.

## International Working Group on Remission of Type 2 Diabetes

Sean McKelvey, Institute for Personalized Therapeutic Nutrition, Vancouver, Canada; William Yancy Jr., Lifestyle and Weight Management Center and Department of Medicine, Duke University, United States; Susan Wolver, General Internal Medicine, Diplomate, American Board of Obesity Medicine, VCU Medical Weight Loss Program, Richmond, Virginia, United States; Campbell Murdoch, Millbrook Surgery, Somerset, United Kingdom; Brian Lenzkes, Internal Medicine San Diego CA, United States; Caroline Roberts, Virta Health, United States; David Cavan, Educator and Author, United Kingdom; David Unwin, The Norwood Surgery, Southport, United Kingdom; Eric Westman, Department of Medicine, Duke University, United States; Miriam Berchuk, Dipl American Board of Obesity Medicine, Canada; Graham Phillips, United Kingdom; Ali Irshad Al Lawati, Internal Medicine, Diwan of Royal Court, Oman; Nafeeza Hj Mohd Ismail, School of Medicine, International Medical University, Malaysia; Daniel Katambo, Afyaplanet, Kenya; Anne-Sophie Brazeau, McGill University, Canada.

## Conflict of Interest

The authors declare that the research was conducted in the absence of any commercial or financial relationships that could be construed as a potential conflict of interest.

## Publisher's Note

All claims expressed in this article are solely those of the authors and do not necessarily represent those of their affiliated organizations, or those of the publisher, the editors and the reviewers. Any product that may be evaluated in this article, or claim that may be made by its manufacturer, is not guaranteed or endorsed by the publisher.

## Appendix 1: Current published clinical guidelines on LCD with medication reduction

- Guideline Central: Low-Carbohydrate Nutrition Approaches in Patients with Obesity, Prediabetes and Type 2 Diabeteshttp://eguideline.guidelinecentral.com/i/1180534-low-carb-nutritional-approaches-guidelines-advisory/0?
-UK version http://eguideline.guidelinecentral.com/i/1183584-low-carb-nutrition-queens-units/0?
- Adapting diabetes medication for low carbohydrate management of type 2 diabetes: a practical guidehttps://bjgp.org/content/69/684/360
- A clinician's guide to inpatient low carbohydrate diets for remission of type 2 diabetes: toward a standard of care protocolhttps://www.openaccessjournals.com/articles/a-clinicians-guide-to-inpatient-lowcarbohydrate-diets-for-remission-of-type-2-diabetes-toward-a-standard-of-care-protocol-12898.html
- Clinical Guidelines For the Prescription of Carbohydrate Restriction as a Therapeutic Intervention/Low Carb USA International Scientific and Clinical Advisoryhttps://www.lowcarbusa.org/standard-of-care/clinical-guidelines/
